# Correction: Expression of CGRP in the Trigeminal Ganglion and Its Effect on the Polarization of Macrophages in Rats with Temporomandibular Arthritis

**DOI:** 10.1007/s10571-024-01527-9

**Published:** 2025-01-09

**Authors:** Junli Tao, Xiaohui Wang, Jie Xu

**Affiliations:** 1 College of Stomatology, Chongqing Medical University, Chongqing, China; 2 Chongqing Key Laboratory for Oral Diseases and Biomedical Sciences, Chongqing, China; 3 Chongqing Municipal Key Laboratory of Oral Biomedical Engineering of Higher Education, Chongqing, China

**Correction to: Cellular and Molecular Neurobiology (2024) 44:22** 10.1007/s10571-024-01456-7

The original version of this article unfortunately contained an error in figures.

In Figs. 5 and 6, there occurred a partial duplication of images in Figs. 5A and 6A as the authors have inadvertently used the enlarged and cropped images in the article.

The incorrect version of Fig. 5.


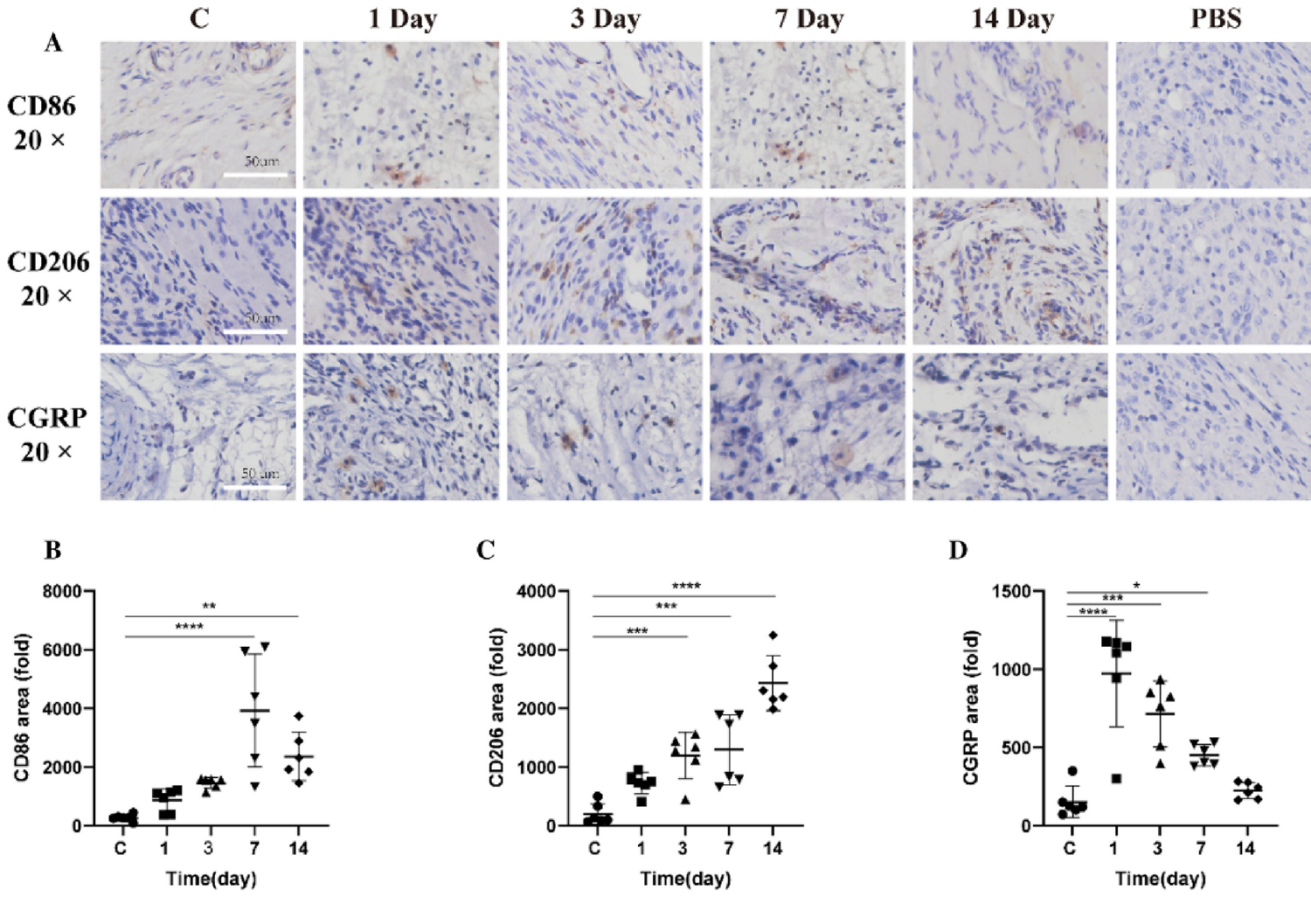


The correct version of Fig. 5.

**Fig. 5:**
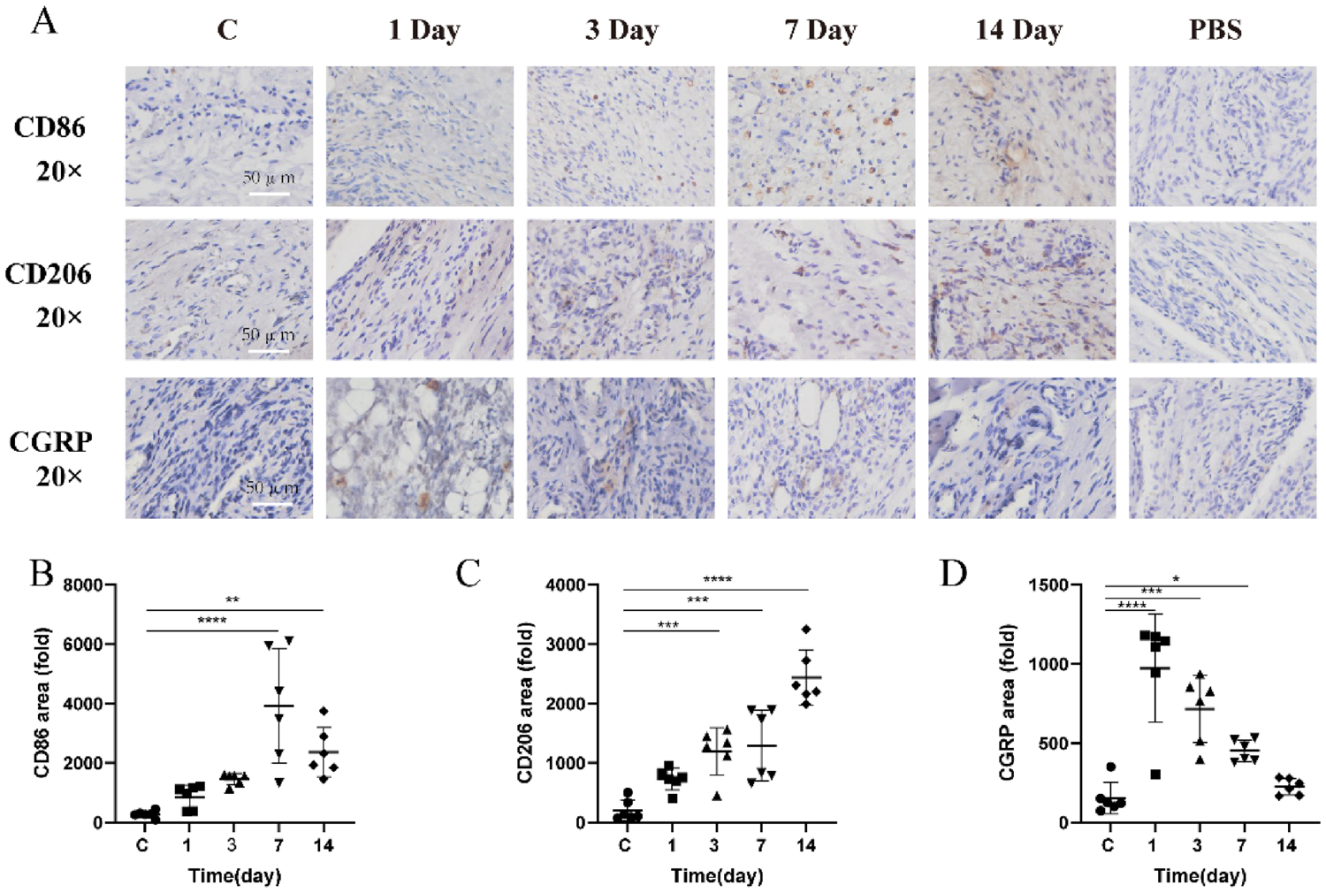
Temporal dynamics and distribution of CD86, CD206, and CGRP in the temporomandibular joint's soft tissue post-CFA induced inflammation. **A** Depicts immunohistochemical localization of CD86, CD206, and CGRP. **B** Highlights CD86 expression. **C** Details CD206 expression. **D** Illustrates CGRP expression. Data are presented as mean ± SD, analyzed via one-way ANOVA with Dunnett’s *t* post hoc test. Sample size (*n*) = 6. NTR = 3. Notations of significance: *p < 0.05, **p < 0.01, ***p < 0.001, ****p < 0.0001

The incorrect version of Fig. 6.


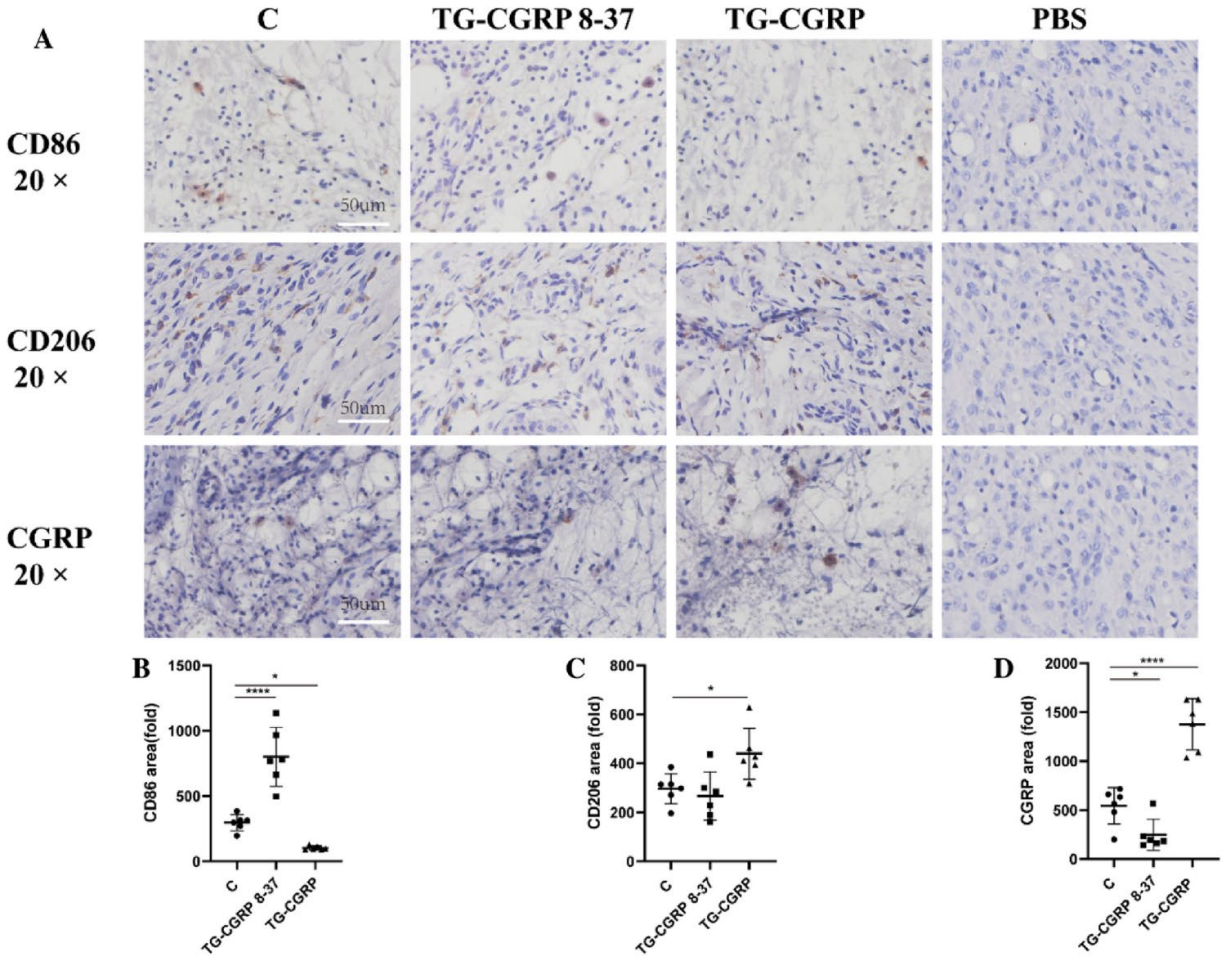


The correct version of Fig. 6.

**Fig. 6:**
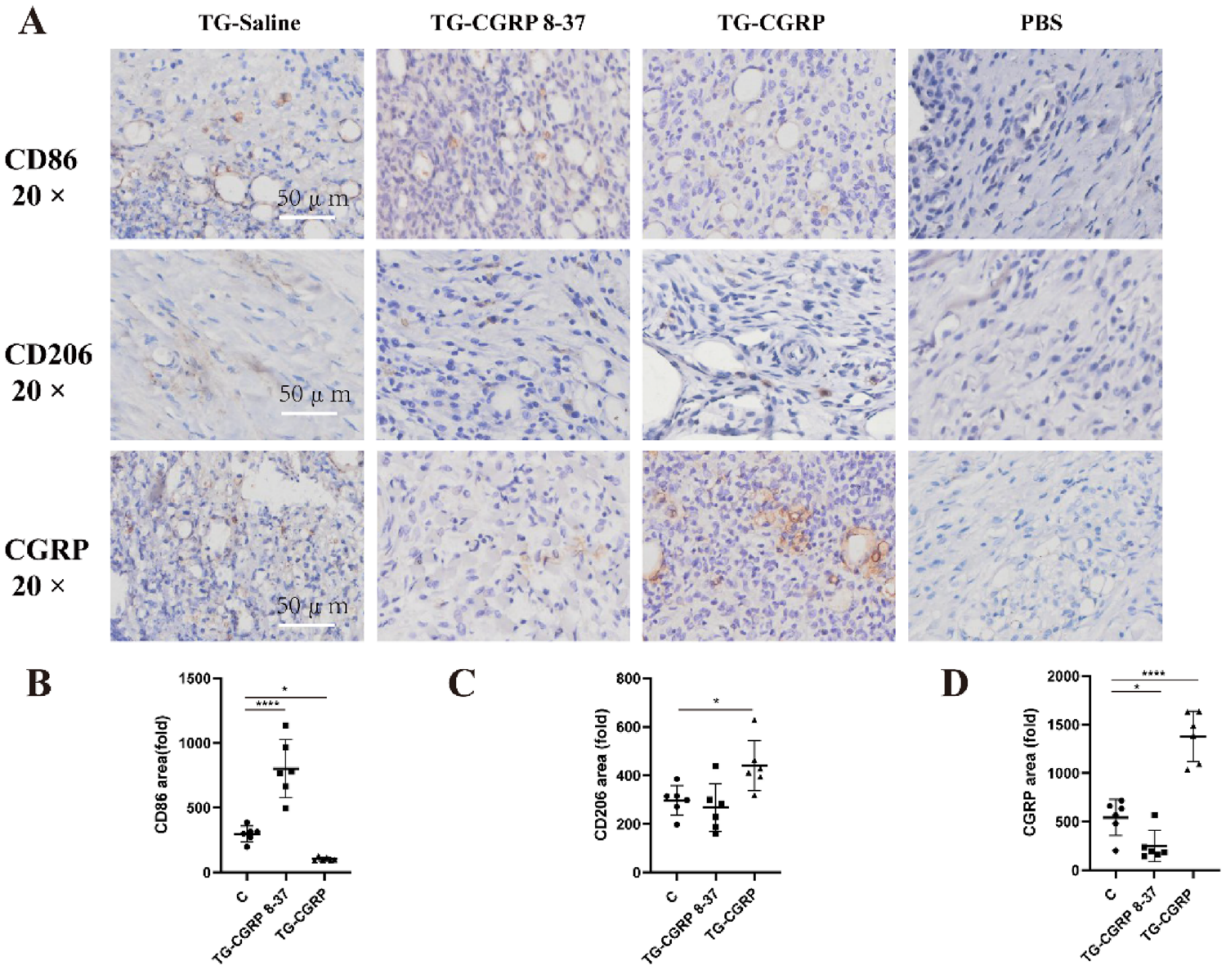
Influence of IG CGRP and CGRP 8–37 on CD86/CD206/CGRP in TMJ soft tissue: **A** Immunohistochemistry for CD86/CD206/CGRP, **B** changes in CD86 post-injection, **C** CD206 expression shifts, and **D** CGRP protein alterations post-treatment. Data are represented as means ± SD and were analyzed by one-way analysis of variance (ANOVA) followed by Dunnett’s *t* test. Sample size (*n*) = 6; NTR = 3. Significance levels indicated as: *p < 0.05, **p < 0.01, ***p < 0.001, ****p < 0.0001

The original article has been corrected.

